# Active fluidics at lower intraocular pressure reduces intraoperative discomfort during phacoemulsification: a propensity score-matched cohort study

**DOI:** 10.3389/fmed.2026.1783229

**Published:** 2026-04-22

**Authors:** Yuanjiao Qiao, Ye Ye, Fangyan Liu, Lishi Luo, Xiaosheng Huang, Biyun Liang, Wenqun Xi, Xinhua Liu, Kun Zeng

**Affiliations:** Department of Cataract, Shenzhen Eye Hospital, Shenzhen Eye Medical Center, Southern Medical University, Xiangmihu Subdistrict, Futian District, Shenzhen, Guangdong, China

**Keywords:** active fluidics, corneal edema, intraocular pressure, intraoperative discomfort, phacoemulsification

## Abstract

**Background:**

Phacoemulsification under topical anesthesia requires adequate intraocular pressure (IOP) maintenance, but elevated IOP may contribute to patient discomfort and corneal stress. Active fluidics technology enables surgery at lower target pressures, yet the impact on patient-centered outcomes remains underexplored.

**Methods:**

This prospective cohort study compared the active fluidics system (target IOP 30 mmHg) with the gravity-based fluidics system (approximately 62 mmHg) in patients undergoing phacoemulsification for age-related cataract. Propensity score matching (1:1) balanced baseline characteristics between groups. The primary outcome was intraoperative discomfort, assessed by supplemental anesthesia requirement and patient-reported pain scores (numerical rating scale, 0–10). Secondary outcomes included central corneal thickness (CCT), endothelial cell density (ECD), and visual acuity over 1 month of follow-up.

**Results:**

After matching, 130 patients (65 per group) were analyzed. Fewer patients in the Active Fluidics Group required supplemental anesthesia (3.1% vs. 13.8%; relative risk 0.22; 95% CI 0.05–0.98; *p* = 0.028). Median pain scores during phacoemulsification were lower in the Active Fluidics Group (1 vs. 3; *p* < 0.001). Day-1 CCT increase was smaller with active fluidics (28.4 vs. 46.8 μm; mean difference −18.4 μm; 95% CI −26.1 to −10.7; *p* < 0.001), though this difference resolved by 1 month. ECD loss and final visual acuity were comparable between groups. No intraoperative complications occurred.

**Conclusion:**

Phacoemulsification using active fluidics at lower target IOP was associated with reduced intraoperative discomfort and attenuated early postoperative corneal edema while maintaining equivalent safety and visual outcomes.

## Introduction

Cataract remains a leading cause of treatable blindness globally, accounting for approximately 45% of visual impairment among adults over 50 years of age (1). Phacoemulsification with intraocular lens implantation is the standard surgical treatment, with millions of procedures performed annually worldwide. While contemporary cataract surgery achieves excellent visual outcomes in most cases, optimizing patient comfort and minimizing corneal stress during surgery remain important goals, particularly as patient expectations continue to rise (2, 3).

During phacoemulsification, adequate intraocular pressure (IOP) must be maintained to stabilize the anterior chamber and facilitate safe lens removal. Conventional gravity-based fluidics systems achieve this by elevating the irrigation bottle, typically generating IOPs of 60–90 mmHg depending on bottle height (4, 5). However, elevated IOP during surgery may contribute to patient discomfort under topical anesthesia and increase mechanical stress on the corneal endothelium (6, 7). These factors have prompted interest in surgical techniques that maintain anterior chamber stability at lower pressure levels.

Active fluidics technology represents an evolution in phacoemulsification system design. Unlike gravity-based systems, active fluidics platforms use integrated pressure sensors and microprocessor-controlled pumps to monitor and regulate IOP in real time (4, 5). This technology enables surgery at lower target pressures (as low as 30 mmHg) while maintaining chamber stability through dynamic compensation for fluid outflow during phacoemulsification (6). The Centurion Vision System with Active Sentry handpiece (Alcon Laboratories) exemplifies this approach, incorporating an integrated pressure sensor in the handpiece that provides real-time pressure feedback.

Several studies have compared active and gravity-based fluidics systems. Laboratory models have demonstrated improved anterior chamber stability with active fluidics during simulated post-occlusion surge (6). Clinical studies have reported reduced fluid consumption and cumulative dissipated energy with active fluidics platforms (7, 8). A meta-analysis of randomized trials found modest reductions in corneal thickness increase and anterior chamber inflammation with active fluidics systems (8). However, most comparative studies have focused on surgical efficiency metrics rather than patient-centered outcomes such as intraoperative comfort.

The relationship between intraoperative IOP and patient discomfort during topical anesthesia cataract surgery has received limited investigation. Hou et al. (9) reported that lowering irrigation bottle height reduced discomfort during phacoemulsification, suggesting that IOP may influence pain perception. Whether the lower operating pressures achievable with active fluidics systems translate to meaningful improvements in patient comfort has not been systematically evaluated.

This prospective cohort study compared clinical outcomes between two contemporary phacoemulsification platforms: the Centurion system utilizing active fluidics at 30 mmHg target IOP, and the Stellaris system utilizing gravity-based fluidics at approximately 62 mmHg. Propensity score matching was employed to balance baseline characteristics between groups. We focused on intraoperative discomfort as the primary outcome, with secondary assessment of early postoperative corneal changes over 1 month of follow-up. We hypothesized that surgery using the active fluidics platform would be associated with reduced intraoperative discomfort and less early postoperative corneal edema.

## Methods

### Study design and setting

This prospective, comparative cohort study was conducted at Shenzhen Eye Hospital between January 2024 and August 2024. The study compared clinical outcomes between two phacoemulsification platforms: the Centurion Vision System with Active Sentry handpiece (Alcon Laboratories, Fort Worth, TX, USA), utilizing active fluidics with real-time intraocular pressure monitoring, and a commercially available gravitybased fluidics system from another manufacturer. The study protocol was approved by the Institutional Ethics Committee of Shenzhen Eye Hospital and adhered to the tenets of the Declaration of Helsinki. All participants provided written informed consent.

### Participants

We enrolled consecutive patients presenting for elective cataract surgery who met the following inclusion criteria: (1) age 50–75 years; (2) diagnosis of age-related cataract graded II–III on the Lens Opacity Classification System III (LOCS III); (3) corneal endothelial cell density (ECD) >1,500 cells/mm^2^; (4) axial length 22–26 mm; and (5) scheduled for primary unilateral phacoemulsification (first-eye surgery only) with posterior chamber intraocular lens implantation.

Exclusion criteria included: (1) previous ocular surgery or trauma; (2) coexisting ocular pathology (glaucoma, uveitis, diabetic retinopathy, macular degeneration, corneal opacity, or pseudoexfoliation syndrome); (3) anterior chamber depth < 2.5 mm; (4) poorly dilating pupil (< 6 mm after pharmacological dilation); (5) systemic conditions affecting pain perception (fibromyalgia, diabetic neuropathy, chronic pain syndrome, or current use of analgesic medications); (6) cognitive impairment precluding reliable pain assessment; or (7) inability to attend scheduled follow-up visits.

### Group assignment and matching

Patients were assigned to surgical platforms based on operating room scheduling: those operated on Mondays and Wednesdays received surgery using the Centurion system (Active Fluidics Group), while those operated on Tuesdays and Thursdays received surgery using the Stellaris system (Gravity Fluidics Group). This scheduling-based allocation minimized selection bias while reflecting real-world clinical practice.

To address potential confounding from baseline differences, we employed 1:1 propensity score matching. Propensity scores were calculated using a logistic regression model incorporating age, sex, LOCS III nuclear grade, preoperative best-corrected distance visual acuity (BCDVA), central corneal thickness (CCT), ECD, axial length, and preoperative intraocular pressure (IOP). Patients were matched using nearest-neighbor matching with a caliper width of 0.2 standard deviations of the logit of the propensity score. Covariate balance after matching was assessed using standardized mean differences (SMD), with SMD < 0.1 indicating adequate balance.

### Surgical technique

All surgeries were performed by two experienced surgeons (Surgeon A and Surgeon B), each with >500 annual phacoemulsification procedures and proficiency with both platforms. To control for surgeon-related variability, each surgeon performed equal numbers of procedures with each system, alternating weekly between platforms. The 30 mmHg target IOP for the Centurion system was selected based on manufacturer guidelines and prior studies demonstrating anterior chamber stability, safety, and reduced corneal stress at this physiologic level (4, 10).

***Preoperative preparation***

Pupils were dilated with tropicamide 1% and phenylephrine 2.5% eye drops. Topical anesthesia was achieved with proparacaine 0.5% applied at 10, 5, and 1 min before surgery. Supplemental intracameral preservative-free lidocaine 1% (0.3 ml) was administered at the start of each procedure.

***Centurion active fluidics group***

The Centurion system was configured with target IOP set at 30 mmHg using the Active Sentry handpiece, which provides real-time IOP monitoring through an integrated pressure sensor. Phacoemulsification was performed using the Ozil Intelligent Phaco (IP) torsional mode with the following parameters: vacuum 400 mmHg, aspiration flow rate 30 cc/min, and bottle height equivalent maintained dynamically by the active fluidics system.

***Stellaris gravity fluidics group***

The Stellaris system was configured with an irrigation bottle height of 85 cm (corresponding to approximately 62 mmHg IOP) and vacuum set at 400 mmHg (7). Aspiration flow was controlled indirectly via the vacuum-based Venturi pump and foot pedal input, without a direct surgeon-set aspiration flow rate parameter.

***Standardized surgical steps***

All procedures employed a 2.4-mm temporal clear corneal incision, continuous curvilinear capsulorhexis (5.0–5.5 mm diameter), hydrodissection, and divide-and-conquer or stop-and-chop nucleofractis technique based on nuclear density. Cortical removal was performed using bimanual irrigation/aspiration. A single-piece hydrophobic acrylic IOL (AcrySof IQ SN60WF, Alcon) was implanted in all cases. Viscoelastic (sodium hyaluronate 1.7%, Healon GV) was used for anterior chamber maintenance and completely removed at procedure completion.

### Outcome measures

***Primary outcome***

Intraoperative discomfort, assessed by: (a) the proportion of patients requiring supplemental topical anesthesia (proparacaine 0.5%) during phacoemulsification due to expressed discomfort, and (b) patient-reported pain using a validated 11-point numerical rating scale (NRS; 0 = no pain, 10 = worst imaginable pain) administered immediately postoperatively for three surgical phases: phacoemulsification, irrigation/aspiration, and IOL implantation.

### Secondary outcomes

1. Central corneal thickness (CCT) measured by anterior segment optical coherence tomography (AS-OCT; Casia2, Tomey Corporation, Nagoya, Japan).
2. Corneal endothelial cell density (ECD) and morphology (coefficient of variation [CV], percentage of hexagonal cells) measured by specular microscopy (SP-3000P, Topcon Corporation, Tokyo, Japan).
3. BCDVA measured using Early Treatment Diabetic Retinopathy Study (ETDRS) charts and converted to logMAR.
4. IOP measured by Goldmann applanation tonometry.

***Intraoperative parameters***

Total surgical time, phacoemulsification time, cumulative dissipated energy (CDE; %-seconds) for Centurion or average ultrasound power (%) × phacoemulsification time (seconds) for Stellaris [approximated as CDE equivalent using methods from Vasavada et al. (2) and Luo et al. (7), acknowledging platform-specific calculation differences], estimated fluid usage, and occurrence of intraoperative complications.

***Assessment timepoints***

Preoperative (within 7 days of surgery), postoperative day 1, week 1, and month 1.

***Safety outcomes***

Intraoperative complications (posterior capsule rupture, zonular dehiscence, iris trauma, Descemet membrane detachment) and postoperative complications (corneal edema grade ≥2, IOP spike >25 mmHg, cystoid macular edema, endophthalmitis, IOL dislocation).

### Masking

Outcome assessors performing postoperative measurements (AS-OCT, specular microscopy, visual acuity, tonometry) were masked to group assignment. The statistician performing data analysis was also masked. Due to the nature of the intervention, surgeons and patients could not be masked.

### Sample size calculation

Based on preliminary data and published literature, we estimated that supplemental anesthesia would be required in 15% of gravity fluidics cases and 3% of active fluidics cases. To detect this difference with 80% power and two-sided α of 0.05, 58 patients per group were required (chi-square test). Anticipating 10% loss to follow-up and the need for propensity score matching, we aimed to enroll 80 patients per group (160 total).

### Statistical analysis

Continuous variables were expressed as mean ± standard deviation (SD) or median [interquartile range (IQR)] based on distribution normality assessed by Shapiro–Wilk test. Categorical variables were expressed as frequencies and percentages. Between-group comparisons used independent *t*-tests or Mann–Whitney *U*-tests for continuous variables and chi-square or Fisher's exact tests for categorical variables, as appropriate.

Within-group changes over time were analyzed using repeated-measures ANOVA with Greenhouse–Geisser correction for sphericity violations. To account for multiple comparisons across secondary outcomes, we applied the Benjamini–Hochberg procedure to control the false discovery rate (FDR) at 5%.

Multivariable linear regression was used to identify factors associated with postoperative CCT change, adjusting for age, nuclear grade, phacoemulsification time, CDE, and baseline CCT. A sensitivity analysis was performed using the unmatched cohort with inverse probability of treatment weighting (IPTW) to verify robustness of findings.

All analyses were performed using R version 4.3.1 (R Foundation for Statistical Computing, Vienna, Austria) with the MatchIt, tableone, and emmeans packages. Two-sided *p* < 0.05 was considered statistically significant for the primary outcome; FDR-adjusted *p* < 0.05 was used for secondary outcomes.

## Results

### Participant flow and baseline characteristics

**Figure 1:**
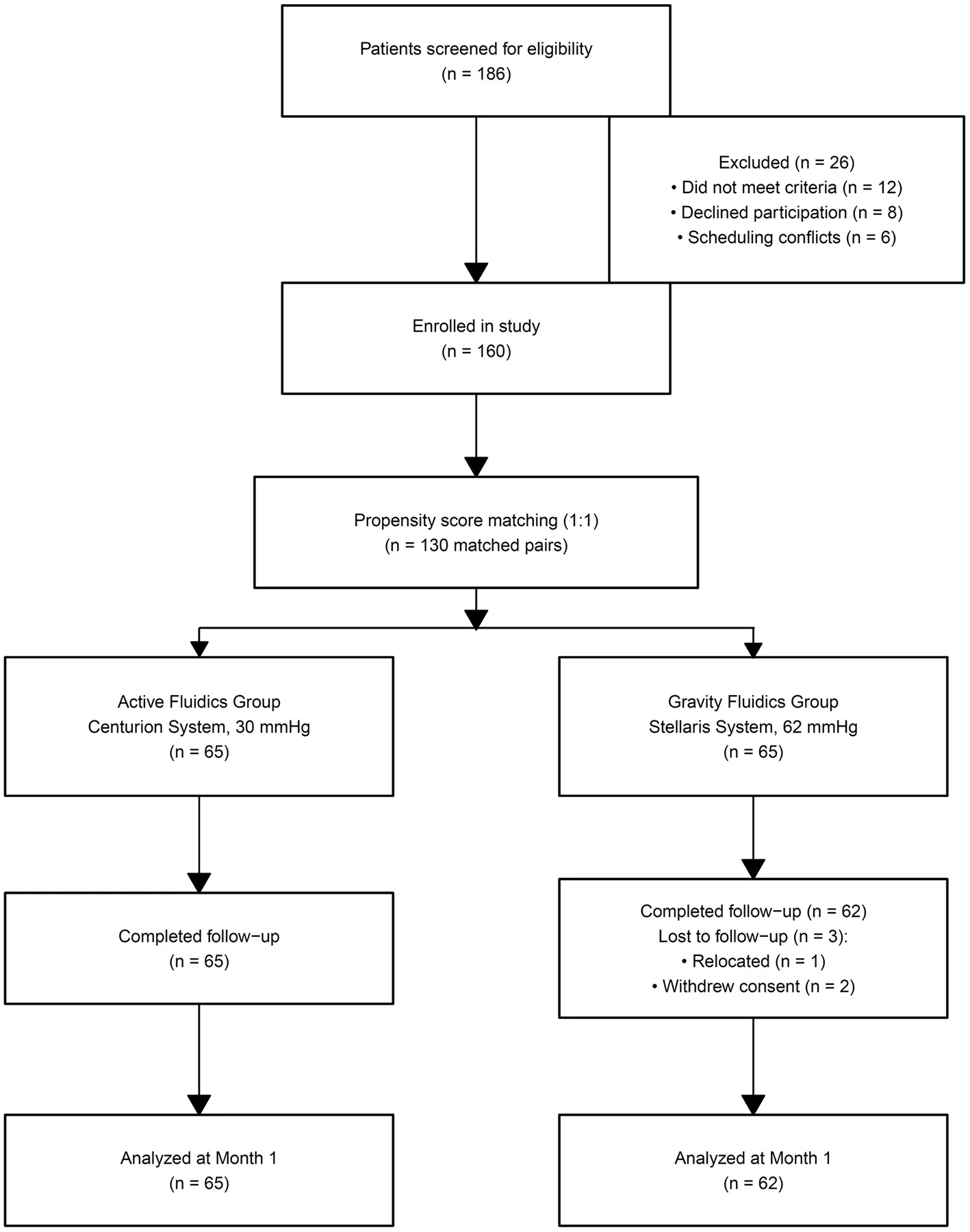
Study flow diagram. Flow diagram showing patient screening, enrollment, propensity score matching, and follow-up. Of 186 patients screened, 160 were enrolled and 130 included after 1:1 propensity score matching (65 per group). Three patients in the Gravity Fluidics Group were lost to follow-up at month 1.

Between January and August 2024, 186 patients were screened for eligibility. Twenty-six patients were excluded (12 did not meet inclusion criteria, eight declined participation, six had scheduling conflicts). Of the 160 enrolled patients, propensity score matching yielded 65 well-matched pairs (130 patients total; Figure 1). Three patients in the Gravity Fluidics Group were lost to follow-up at month 1 (1 relocated, 2 withdrew consent), resulting in 65 and 62 patients for the final 1-month analysis.

After propensity score matching, baseline characteristics were well-balanced between groups (all SMD < 0.1; Table 1). Mean age was 65.2 ± 6.8 years in the Active Fluidics Group and 65.8 ± 6.4 years in the Gravity Fluidics Group. Nuclear cataract grade distribution, preoperative visual acuity, corneal parameters, and ECD were comparable (Table 1).

**Table 1:** Baseline characteristics after propensity score matching.

Characteristic	Active fluidics group (*n* = 65)	Gravity fluidics group (*n* = 65)	SMD
Age, years	65.2 ± 6.8	65.8 ± 6.4	0.09
Female sex, *n* (%)	38 (58.5)	36 (55.4)	0.06
Right eye, *n* (%)	34 (52.3)	32 (49.2)	0.06
Nuclear grade (LOCS III)			0.04
Grade II, *n* (%)	28 (43.1)	27 (41.5)	
Grade III, *n* (%)	37 (56.9)	38 (58.5)	
Preoperative BCDVA, logMAR	0.52 ± 0.24	0.54 ± 0.26	0.08
Central corneal thickness, μm	536.4 ± 28.6	538.2 ± 30.4	0.06
Endothelial cell density, cells/mm^2^	2,724 ± 286	2,698 ± 302	0.09
Coefficient of variation, %	32.4 ± 6.2	33.1 ± 6.8	0.08
Hexagonal cells, %	58.6 ± 8.4	57.8 ± 8.8	0.09
Axial length, mm	23.62 ± 1.08	23.58 ± 1.12	0.04
Anterior chamber depth, mm	3.12 ± 0.32	3.08 ± 0.34	0.07
Preoperative IOP, mmHg	14.2 ± 2.4	14.4 ± 2.6	0.08
Surgeon A, *n* (%)	33 (50.8)	32 (49.2)	0.03

Data presented as mean ± SD or *n* (%). SMD, standardized mean difference; LOCS, lens opacity classification system; BCDVA, best-corrected distance visual acuity; IOP, intraocular pressure. SMD < 0.1 indicates adequate balance.

### Primary outcome: intraoperative discomfort

Significantly fewer patients in the Active Fluidics Group required supplemental intraoperative anesthesia compared to the Gravity Fluidics Group (3.1% [2/65] vs. 13.8% [9/65]; relative risk 0.22; 95% CI 0.05–0.98; *p* = 0.028; Table 2).

**Table 2:** Primary outcome: intraoperative discomfort.

Outcome	Active fluidics (*n* = 65)	Gravity fluidics (*n* = 65)	Effect estimate (95% CI)	*p*-value
Supplemental anesthesia required				
*n* (%)	2 (3.1)	9 (13.8)	RR 0.22 (0.05–0.98)	0.028
NRS pain scores, median (IQR)				
Phacoemulsification	1 (0–2)	3 (2–4)	–	< 0.001
Irrigation/aspiration	1 (0–2)	2 (1–3)	–	< 0.001
IOL implantation	1 (0–1)	1 (1–2)	–	0.042
Overall (mean of 3 phases)	1.0 ± 0.8	2.2 ± 1.0	MD −1.2 (−1.5 to −0.9)	< 0.001

RR, relative risk; MD, mean difference; NRS, numerical rating scale (0–10); IQR, interquartile range; IOL, intraocular lens.

Patient-reported pain scores were significantly lower in the Active Fluidics Group across all surgical phases. During phacoemulsification, median NRS scores were 1 (IQR 0–2) vs. 3 (IQR 2–4; *p* < 0.001). During irrigation/aspiration, scores were 1 (IQR 0–2) vs. 2 (IQR 1–3; *p* < 0.001). During IOL implantation, scores were 1 (IQR 0–1) vs. 1 (IQR 1–2; *p* = 0.042; Table 2).

### Secondary outcomes

Central Corneal Thickness: Postoperative CCT increased in both groups, with significantly less increase in the Active Fluidics Group at day 1 (Table 3). Mean CCT change from baseline was +28.4 ± 18.2 μm (5.3% ± 3.4%) in the Active Fluidics Group vs. +46.8 ± 26.4 μm (8.7% ± 4.9%) in the Gravity Fluidics Group (mean difference −18.4 μm; 95% CI −26.1 to −10.7; adjusted *p* < 0.001).

**Table 3:** Corneal outcomes at follow-up timepoints.

Parameter	Timepoint	Active fluidics (*n* = 65)	Gravity fluidics (*n* = 65)	Difference (95% CI)	adjusted p^*^
CCT, μm	Baseline	536.4 ± 28.6	538.2 ± 30.4	–	–
	Day 1	564.8 ± 32.4	585.0 ± 38.6	−20.2 (−32.4 to −8.0)	< 0.001
	Week 1	549.0 ± 29.8	556.4 ± 32.2	−7.4 (−18.2 to 3.4)	0.012
	Month 1^†^	540.6 ± 28.2	544.0 ± 30.8	−3.4 (−13.6 to 6.8)	0.238
CCT change, %	Day 1	5.3 ± 3.4	8.7 ± 4.9	−3.4 (−4.8 to −2.0)	< 0.001
	Week 1	2.4 ± 1.9	3.4 ± 2.4	−1.0 (−1.8 to −0.2)	0.012
	Month 1^†^	0.8 ± 1.2	1.1 ± 1.5	−0.3 (−0.8 to 0.2)	0.238
ECD, cells/mm^2^	Baseline	2,724 ± 286	2,698 ± 302	–	–
	Month 1^†^	2,538 ± 312	2,474 ± 328	64 (−48 to 176)	0.156
ECD loss, %	Month 1^†^	6.8 ± 5.2	8.2 ± 6.1	−1.4 (−3.4 to 0.6)	0.156
CV, %	Month 1^†^	34.8 ± 7.2	35.6 ± 7.8	−0.8 (−3.4 to 1.8)	0.524
Hexagonal cells, %	Month 1^†^	56.2 ± 9.2	55.4 ± 9.6	0.8 (−2.4 to 4.0)	0.612

^*^CCT, central corneal thickness; ECD, endothelial cell density; CV, coefficient of variation. Adjusted for multiple comparisons using Benjamini–Hochberg procedure.

^†^Month 1 analysis includes *n* = 65 active fluidics, *n* = 62 gravity fluidics (3 lost to follow-up).

At week 1, CCT differences persisted but were attenuated: +12.6 ± 10.4 μm (2.4% ± 1.9%) vs. +18.2 ± 12.8 μm (3.4% ± 2.4%; adjusted *p* = 0.012). By month 1, CCT had returned to near-baseline values in both groups with no significant difference (+4.2 ± 6.8 μm vs. +5.8 ± 8.2 μm; adjusted *p* = 0.238).

Corneal Endothelial Cell Density: ECD decreased postoperatively in both groups (Table 3). At month 1, mean ECD loss was −186 ± 142 cells/mm^2^ (6.8% ± 5.2%) in the Active Fluidics Group vs. −224 ± 168 cells/mm^2^ (8.2% ± 6.1%) in the Gravity Fluidics Group. This difference did not reach statistical significance (adjusted *p* = 0.156). Endothelial morphology parameters (CV, hexagonality) showed similar changes between groups (Table 3).

Visual Acuity: BCDVA improved significantly in both groups postoperatively (Table 4). At day 1, mean BCDVA was 0.12 ± 0.10 logMAR in the Active Fluidics Group vs. 0.16 ± 0.12 logMAR in the Gravity Fluidics Group (adjusted *p* = 0.048). By week 1, values were 0.04 ± 0.06 vs. 0.05 ± 0.07 logMAR (adjusted *p* = 0.412), and by month 1, both groups achieved excellent outcomes (0.02 ± 0.04 vs. 0.02 ± 0.05 logMAR; adjusted *p* = 0.864).

**Table 4:** Visual and pressure outcomes.

Parameter	Timepoint	Active fluidics (*n* = 65)	Gravity fluidics (*n* = 65)	Adjusted p^*^
BCDVA, logMAR	Baseline	0.52 ± 0.24	0.54 ± 0.26	–
	Day 1	0.12 ± 0.10	0.16 ± 0.12	0.048
	Week 1	0.04 ± 0.06	0.05 ± 0.07	0.412
	Month 1^†^	0.02 ± 0.04	0.02 ± 0.05	0.864
IOP, mmHg	Baseline	14.2 ± 2.4	14.4 ± 2.6	–
	Day 1	14.8 ± 3.2	15.4 ± 3.8	0.324
	Week 1	13.6 ± 2.8	13.8 ± 2.6	0.682
	Month 1^†^	13.4 ± 2.4	13.6 ± 2.6	0.648

^*^BCDVA, best-corrected distance visual acuity; IOP, intraocular pressure. Adjusted for multiple comparisons.

^†^Month 1: *n* = 65 active fluidics, *n* = 62 gravity fluidics.

Intraocular Pressure: Postoperative IOP did not differ significantly between groups at any timepoint (Table 4). No cases of IOP spike >25 mmHg occurred in either group.

### Intraoperative parameters

Surgical parameters are summarized in Table 5. Mean total surgical time was comparable (8.4 ± 2.1 min vs. 8.8 ± 2.4 min; *p* = 0.312). Phacoemulsification time was similar (38.2 ± 16.4 s vs. 41.6 ± 18.2 s; *p* = 0.268). CDE (or equivalent; %-seconds) was lower in the Active Fluidics Group (8.2 ± 4.6 vs. 10.4 ± 5.8; *p* = 0.016). Estimated fluid usage was significantly lower in the Active Fluidics Group (42.6 ± 12.4 ml vs. 68.4 ± 18.6 ml; *p* < 0.001).

**Table 5:** Intraoperative parameters.

Parameter	Active fluidics (*n* = 65)	Gravity fluidics (*n* = 65)	*p*-value
Total surgical time, min	8.4 ± 2.1	8.8 ± 2.4	0.312
Phacoemulsification time, s	38.2 ± 16.4	41.6 ± 18.2	0.268
CDE (or equivalent)	8.2 ± 4.6	10.4 ± 5.8	0.016
Estimated fluid usage, ml	42.6 ± 12.4	68.4 ± 18.6	< 0.001
Effective phaco time, s	12.4 ± 6.8	14.8 ± 7.6	0.062

CDE, cumulative dissipated energy. CDE equivalent for Stellaris calculated as average power × phacoemulsification time.

### Multivariable analysis

In multivariable linear regression, surgical platform (β = −16.8; 95% CI −24.2 to −9.4; *p* < 0.001), CDE (β = 1.4; 95% CI 0.6 to 2.2; *p* = 0.001), and nuclear grade (β = 8.2; 95% CI 2.4 to 14.0; *p* = 0.006) were independently associated with day-1 CCT change, after adjusting for age, baseline CCT, and phacoemulsification time (Table 6).

**Table 6:** Multivariable analysis: factors associated with day-1 CCT change.

Variable	β Coefficient	95% CI	*p*-value
Surgical platform (active vs. gravity)	−16.8	−24.2–−9.4	< 0.001
CDE (per unit increase)	1.4	0.6–2.2	0.001
Nuclear grade III (vs. II)	8.2	2.4–14.0	0.006
Age (per year)	0.3	−0.2–0.8	0.248
Baseline CCT (per 10 μm)	0.8	−0.4–2.0	0.186
Phacoemulsification time (per 10 s)	1.2	−0.8–3.2	0.232

Model *R*^2^ = 0.38. CCT, central corneal thickness; CDE, cumulative dissipated energy.

### Subgroup analyses

**Figure 2:**
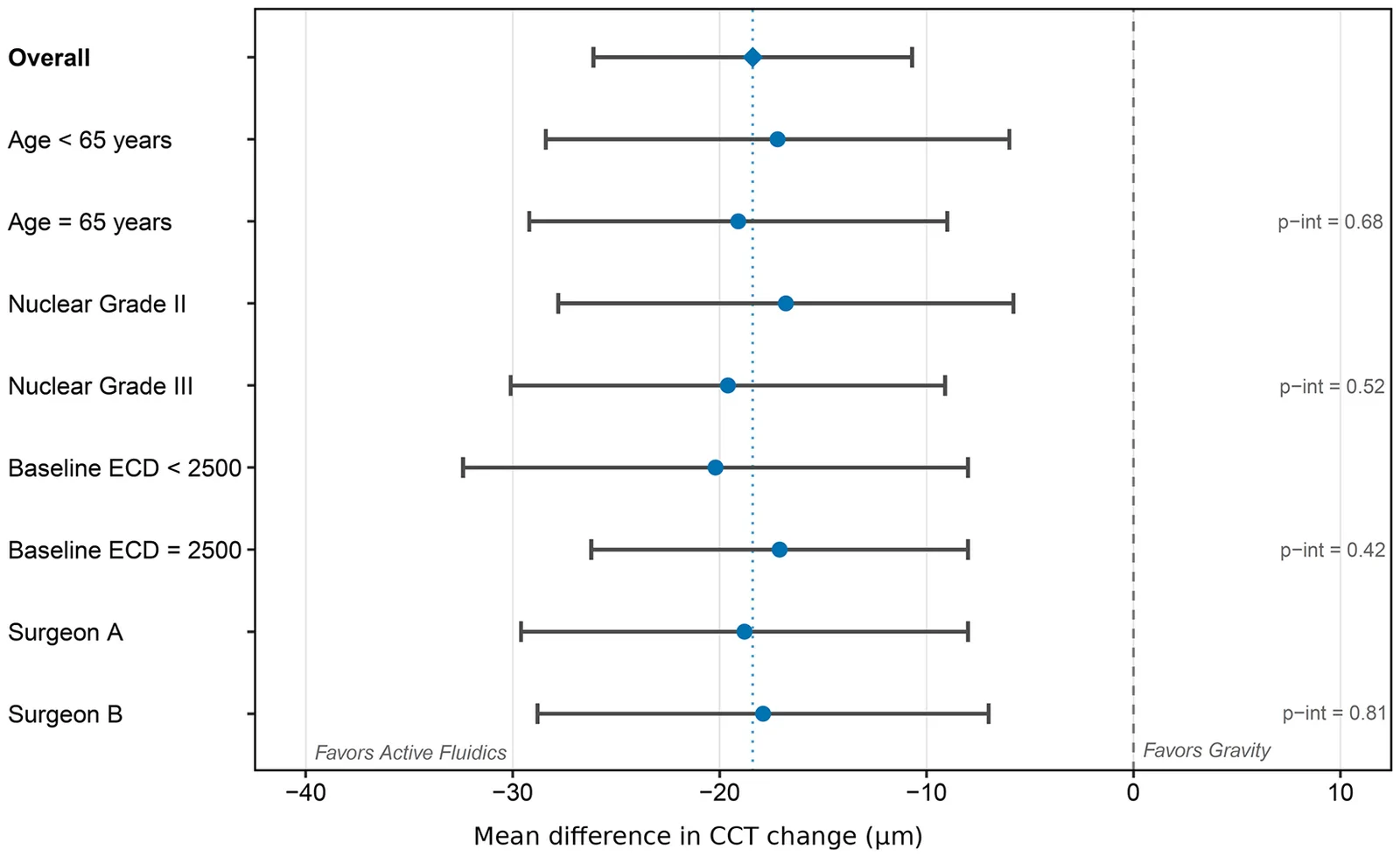
Forest plot of mean difference in day 1 CCT change (active fluidics minus gravity fluidics) across subgroups, with 95% confidence intervals. Negative values favor active fluidics and positive values favor gravity fluidics. *P*-interaction values are shown.

The benefit of active fluidics on day-1 CCT change was consistent across prespecified subgroups (Figure 2), including age (< 65 vs. ≥65 years), nuclear grade (II vs. III), baseline ECD (< 2,500 vs. ≥2,500 cells/mm^2^), and surgeon (A vs. B). Interaction terms were not statistically significant (all *p* > 0.10). Similar analyses for primary outcomes (supplemental anesthesia requirement and median NRS pain scores during phacoemulsification) showed no significant interactions (all *p* > 0.10; Supplementary Table 1), with no surgeon-related differences in outcomes (*p* > 0.50 for all comparisons).

### Sensitivity analyses

Results using IPTW in the unmatched cohort were consistent with the primary analysis. The supplemental anesthesia requirement remained significantly different (OR 0.20; 95% CI 0.05–0.82; *p* = 0.025), as did day-1 CCT change (mean difference −17.2 μm; 95% CI −24.8 to −9.6; *p* < 0.001).

### Safety outcomes

No intraoperative complications occurred in either group. One patient in the Gravity Fluidics Group developed grade 2 corneal edema persisting at day 1, which resolved by week 1. No cases of cystoid macular edema, endophthalmitis, or other serious adverse events were observed (Table 7).

**Table 7:** Safety outcomes.

Complication	Active fluidics (*n* = 65)	Gravity fluidics (*n* = 65)	*p*-value
Intraoperative			
Posterior capsule rupture	0	0	–
Zonular dehiscence	0	0	–
Iris trauma	0	0	–
Descemet detachment	0	0	–
Postoperative			
Corneal edema grade ≥2 at day 1	0	1 (1.5%)	1.000
IOP spike >25 mmHg	0	0	–
Cystoid macular edema	0	0	–
Endophthalmitis	0	0	–

IOP, intraocular pressure.

## Discussion

This prospective cohort study found that phacoemulsification using an active fluidics system operating at lower IOP was associated with reduced intraoperative discomfort compared to a gravity-based system. Patients in the Active Fluidics Group required supplemental anesthesia less frequently (3.1% vs. 13.8%) and reported lower pain scores across all surgical phases. Additionally, early postoperative corneal thickness increase was smaller in the Active Fluidics Group, though this difference resolved by 1 month. Visual outcomes and endothelial cell loss were comparable between groups.

The reduced need for supplemental anesthesia and lower pain scores observed with the active fluidics system are clinically meaningful findings. Under topical anesthesia, patient comfort directly affects cooperation and surgical conditions (11–13). Our results align with observations by Hou et al. (9), who identified elevated IOP as a source of discomfort during phacoemulsification. The mechanism likely involves pressure-sensitive nerve endings in the cornea and anterior segment structures; lower IOP may reduce mechanical stimulation of these pathways. Additionally, the torsional ultrasound in the Centurion system generates less heat compared to longitudinal ultrasound in the Stellaris, which may further reduce thermal-induced discomfort (14).

The magnitude of the difference in pain scores—median NRS of 1 vs. 3 during phacoemulsification—is notable. While both values represent mild-to-moderate discomfort, a two-point difference on an 11-point scale may influence patient experience and willingness to undergo second-eye surgery (13). The consistency of this finding across all surgical phases (phacoemulsification, irrigation/aspiration, and IOL implantation) suggests a genuine effect rather than a phase-specific phenomenon.

It is important to acknowledge that the two systems differ in multiple respects beyond target IOP, including fluidics control mechanism, ultrasound modality (torsional vs. longitudinal), and overall system design. We cannot attribute the observed differences solely to IOP; rather, our findings reflect the aggregate effect of using these distinct platforms. This pragmatic comparison reflects real-world surgical decision-making, where surgeons choose between complete systems rather than isolated parameters.

The Active Fluidics Group demonstrated less corneal thickness increase at postoperative day 1 (5.3% vs. 8.7%) and week 1 (2.4% vs. 3.4%). These findings are consistent with prior reports suggesting that lower IOP and reduced ultrasound energy may decrease corneal endothelial stress (10, 15). Recent *ex vivo* laboratory evidence using intact porcine eyes further indicates that surgical fluid pressures (e.g., 60 mmHg sustained or peaks up to 400 mmHg) over durations typical of phacoemulsification do not cause significant acute iatrogenic corneal endothelial cell loss (16). This supports the notion that early postoperative corneal thickness differences in our study may relate more to transient factors than to permanent endothelial damage. Rauen et al. (10) similarly observed improved corneal transparency and reduced anterior chamber inflammation with the Centurion system at physiologic IOP settings.

The transient nature of this difference—resolving by 1 month—suggests that both systems provide adequate corneal protection for routine cataract surgery in eyes with healthy endothelium. However, the early reduction in edema may have practical significance. More rapid resolution of corneal swelling could accelerate visual recovery in the immediate postoperative period, which is when patients are most aware of their surgical outcome (8). This may be particularly relevant for patients requiring rapid visual rehabilitation or those undergoing sequential bilateral surgery.

Endothelial cell loss at 1 month was numerically lower in the Active Fluidics Group (6.8% vs. 8.2%) but did not reach statistical significance. The 1.4% absolute difference, while not significant in our sample, could be clinically relevant if sustained over time, particularly in patients with compromised baseline endothelial function. Longer follow-up would be needed to determine whether initial differences in corneal stress translate to meaningful differences in long-term endothelial health.

Despite operating at substantially lower IOP, the active fluidics system maintained comparable surgical times, indicating that reduced pressure did not compromise efficiency. The lower cumulative dissipated energy observed with the Centurion system is consistent with the efficiency of torsional ultrasound, which has been shown to reduce energy requirements compared to longitudinal modalities (14, 17). Reduced fluid consumption (42.6 vs. 68.4 ml) reflects the more efficient fluid management of active fluidics technology and may contribute to anterior chamber stability (18).

In multivariable analysis, surgical platform, CDE, and nuclear grade were independently associated with day-1 corneal thickness change. This suggests that both the choice of system and intraoperative energy delivery contribute to early postoperative corneal status. However, caution is warranted in interpreting CDE differences, as platform-specific algorithms may limit direct comparability

These findings have practical implications for surgical decision-making. For most patients undergoing routine cataract surgery, both systems provide safe and effective treatment with excellent visual outcomes. However, active fluidics technology may offer advantages in specific scenarios: patients with anxiety about surgery or low pain tolerance may benefit from the improved comfort profile; patients prioritizing rapid visual recovery may benefit from reduced early corneal edema; and patients with borderline endothelial function may benefit from reduced surgical stress, though this requires further study.

The absence of complications in either group confirms that both platforms maintain adequate surgical safety in appropriately selected patients (19). Surgeons should weigh these potential benefits against practical considerations such as equipment availability, cost, and personal experience with each platform.

This study has several strengths, including prospective design, propensity score matching to balance confounders, use of two experienced surgeons alternating between platforms to control for surgeon-related variability, masked outcome assessment, and standardized surgical protocols. Additionally, restricting to first-eye surgery minimizes bias from heightened pain perception in second-eye procedures (13). However, limitations warrant consideration. First, this was a single-center study with scheduling-based group assignment. While propensity score matching addressed measured confounders, unmeasured differences between groups may exist. Second, surgeons and patients could not be masked to the surgical system, introducing potential bias in subjective outcomes such as pain scores. We mitigated this by masking outcome assessors and using standardized pain assessment protocols.

Third, the two systems differ in multiple technical aspects, precluding attribution of outcomes to any single factor such as IOP. Our comparison reflects the aggregate effect of each platform, which is clinically relevant but limits mechanistic interpretation. Fourth, follow-up was limited to 1 month; longer observation would be needed to assess sustained differences in visual outcomes or endothelial health.

Fifth, we enrolled only patients with moderate cataracts and healthy corneas. Results may differ in more complex cases, such as dense nuclear cataracts, Fuchs dystrophy, or eyes with prior surgery. Finally, our sample size provided adequate power for the primary outcome but may have been insufficient to detect smaller differences in secondary outcomes such as endothelial cell loss.

Further research should address several questions raised by this study. Multicenter trials with diverse surgical settings would enhance generalizability. Longer follow-up extending to 1 year or beyond would clarify whether early corneal differences have lasting implications. Studies specifically enrolling patients with compromised endothelial function could determine whether active fluidics offers meaningful protection in higher-risk eyes (20). Finally, cost-effectiveness analyses comparing the platforms would inform adoption decisions, particularly in resource-limited settings.

Investigation of the specific contribution of IOP vs. other system differences (ultrasound modality, fluidics control) would require controlled experiments isolating individual parameters, which may be feasible in laboratory models but challenging in clinical practice.

## Conclusion

In this prospective cohort study, phacoemulsification using an active fluidics system at lower target IOP was associated with reduced intraoperative discomfort and less early postoperative corneal thickness increase compared to a gravity-based system. Visual outcomes were excellent and comparable between groups. These findings suggest that active fluidics technology may offer patient-centered benefits, particularly regarding surgical comfort, while maintaining equivalent safety and efficacy. The choice between platforms should consider individual patient factors, surgeon experience, and practical constraints.

## Data Availability

The original contributions presented in the study are included in the article/Supplementary material, further inquiries can be directed to the corresponding author.
