# Correction: Active fluidics at lower intraocular pressure reduces intraoperative discomfort during phacoemulsification: a propensity score-matched cohort study

**DOI:** 10.3389/fmed.2026.1959957

**Published:** 2026-08-28

**Authors:** Yuanjiao Qiao, Ye Ye, Fangyan Liu, Lishi Luo, Xiaosheng Huang, Biyun Liang, Wenqun Xi, Xinhua Liu, Kun Zeng

**Affiliations:** Department of Cataract, Shenzhen Eye Hospital, Shenzhen Eye Medical Center, Southern Medical University, Xiangmihu Subdistrict, Futian District, Shenzhen, Guangdong, China

**Keywords:** active fluidics, corneal edema, intraocular pressure, intraoperative discomfort, phacoemulsification

In the published article, there was an error in the **Abstract**, *Methods*. The gravity-based fluidics comparator was incorrectly identified as the “Stellaris Elite System.” This has been corrected to read:

“This prospective cohort study compared the active fluidics system (target IOP 30 mmHg) with the gravity-based fluidics system (approximately 62 mmHg) in patients undergoing phacoemulsification for age-related cataract.”

In the published article, there was an error in the **Introduction**, paragraph 3. The Active Sentry handpiece was incorrectly described as incorporating a “pressure-sensing sleeve.” The relevant component is an integrated pressure sensor in the handpiece. The correct text is:

“The Centurion Vision System with Active Sentry handpiece (Alcon Laboratories) exemplifies this approach, incorporating an integrated pressure sensor in the handpiece that provides real-time pressure feedback.”

In the published article, there was an error in **Methods**, *Study design and setting*, paragraph 1. The gravity-based fluidics comparator was incorrectly identified by a specific system/model designation. The corrected wording removes the comparator manufacturer/model identification while retaining the fluidics method. The correct text is:

“The study compared clinical outcomes between two phacoemulsification platforms: the Centurion Vision System with Active Sentry handpiece (Alcon Laboratories, Fort Worth, TX, USA), utilizing active fluidics with real-time intraocular pressure monitoring, and a commercially available gravity-based fluidics system from another manufacturer.”

The original version of this article has been updated.

