# Supplementary material for: Active fluidics at lower intraocular pressure reduces intraoperative discomfort during phacoemulsification: a propensity score-matched cohort study
Source: Front Med (Lausanne). 2026 Apr 22;13:1783229. doi: 10.3389/fmed.2026.1783229 (PMC13143631; doi:10.3389/fmed.2026.1783229)
Supplement: Supplementary file 1 [file Table_1.docx]

**Supplementary Table 1.** Subgroup Analyses for Primary Outcomes: Interaction p-values and Surgeon Comparison.

| **Subgroup** | **Supplemental Anesthesia (p for interaction)** | **Median NRS Pain (Phaco phase) (p for interaction)** | **Notes / Surgeon p-value** |
| --- | --- | --- | --- |
| Age (<65 vs. ≥65 years) | >0.10 | >0.10 | — |
| Nuclear grade (II vs. III) | >0.10 | >0.10 | — |
| Baseline ECD (<2500 vs. ≥2500 cells/mm²) | >0.10 | >0.10 | — |
| Surgeon (A vs. B) | — | — | p=0.78 (supplemental anesthesia); p=0.64 (pain scores) |

**Note:** Interaction p-values test whether the effect of active fluidics vs. gravity fluidics differed across subgroups. All interactions were non-significant, indicating consistent benefits. Surgeon comparisons were direct between-group tests.
